# Molecular epidemiology of a fatal sarcoptic mange epidemic in endangered San Joaquin kit foxes (*Vulpes macrotis mutica*)

**DOI:** 10.1186/s13071-020-04328-3

**Published:** 2020-09-07

**Authors:** Jaime L. Rudd, Deana L. Clifford, Brian L. Cypher, Joshua M. Hull, A. Jane Riner, Janet E. Foley

**Affiliations:** 1 Department of Medicine and Epidemiology, University of California, Davis, CA 95616 USA; 2 Wildlife Investigations Laboratory, California Department of Fish and Wildlife, Rancho Cordova, CA 95670 USA; 3 Endangered Species Recovery Program, California State University, Stanislaus, Turlock, CA 95382 USA; 4 United States Fish and Wildlife Service, Sacramento, CA 95825 USA

**Keywords:** Bakersfield, California, Management, Host specificity, Microsatellites, Mites, S*arcoptes scabiei*, Wildlife

## Abstract

**Background:**

In 2013, sarcoptic mange, caused by *Sarcoptes scabiei* mites, precipitated a catastrophic decline of the formerly stable urban population of endangered San Joaquin kit foxes (*Vulpes macrotis mutica*) in Bakersfield, California, USA. In 2019, a smaller sarcoptic mange outbreak affected kit foxes 58 km southwest of Bakersfield in the town of Taft, California. To determine whether the Taft outbreak could have occurred as spillover from the Bakersfield outbreak and whether epidemic control efforts must involve not only kit foxes but also sympatric dogs (*Canis lupus familiaris*), coyotes (*Canis latrans*), and red foxes (*Vulpes vulpes*), we evaluated genotypes and gene flow among mites collected from each host species.

**Methods:**

We used 10 *Sarcoptes* microsatellite markers (SARM) to perform molecular typing of 445 *S. scabiei* mites collected from skin scrapings from twenty-two infested kit foxes, two dogs, five coyotes, and five red foxes from Bakersfield, Taft, and other nearby cities.

**Results:**

We identified 60 alleles across all SARM loci; kit fox- and red fox-derived mites were relatively monomorphic, while genetic variability was greatest in Bakersfield coyote- and dog-derived mites. AMOVA analysis documented distinct mite populations unique to hosts, with an overall F_ST_ of 0.467. The lowest F_ST_ (i.e. closest genetic relationship, F_ST_ = 0.038) was between Bakersfield and Taft kit fox-derived mites while the largest genetic difference was between Ventura coyote- and Taft kit fox-derived mites (F_ST_ = 0.843).

**Conclusions:**

These results confirm the close relationship between the Taft and Bakersfield outbreaks. Although a spillover event likely initiated the kit fox mange outbreak, mite transmission is now primarily kit fox-to-kit fox. Therefore, any large-scale population level intervention should focus on treating kit foxes within the city.
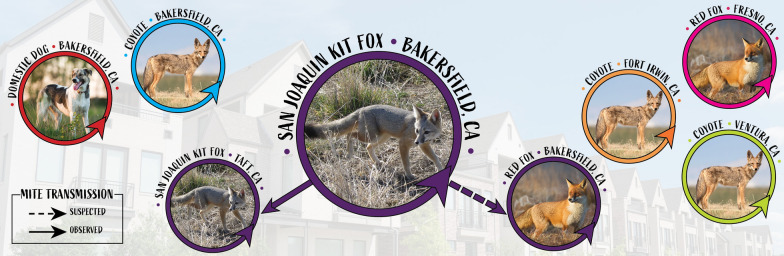

## Background

The first written reference to the skin disease now known as scabies dates to 1200 BCE [1]. From the time of the ancient Greeks and Romans through the middle ages, mange was known as the “itch” [2] and its cause was unknown until 1687, when the Italian physician Giovanni Cosimo Bonomo identified a mite as the causative agent, making scabies one of the first diseases in human history with a known etiology [2]. In 1778, DeGeer formally named the itch mite *Acarus scabiei,* and this classification was revised in 1802 to a new genus, *Sarcoptes,* now with only a single species, *S. scabiei* [1, 3]. The World Health Organization (WHO) describes human scabies as a prevalent, contagious condition affecting more than 200 million people each year worldwide [4].

*Sarcoptes scabiei* is also the agent of sarcoptic mange in animals and affects over 100 mammalian species [3, 5]. Infested domestic animals may have been original sources of mites, which then crossed species boundaries and infested many wildlife hosts [6]. *Sarcoptes scabiei* now appears to be a single but highly variable mite species with many host-restricted genetic variants [7–13]. Although past assessment of host-variant relationships was limited to morphological examination [7], advances in DNA-based techniques now permit improved resolution of those relationships. Microsatellite studies in particular have revealed that, even when multiple species in a community are infested with sarcoptic mange, each may harbor its own host-restricted genetic variant (e.g. host-taxon law) [10]. Documentation of the host specificity of each variant can help determine the host that served as the original source of the epidemic, how many species are involved, and how best to intercede.

A continuing sarcoptic mange epidemic is causing a dramatic decline in a subpopulation of San Joaquin kit foxes (*Vulpes macrotis mutica*, kit fox hereafter). One-hundred years of widespread agricultural and urban development have extirpated these kit foxes throughout much of their historic range, such that this subspecies of kit fox is now federally endangered [14], persisting in a small meta-population of three main subpopulations and less than a dozen satellite subpopulations in the western and southern ends of the San Joaquin Valley in central California [14, 15]. Remarkably, the largest satellite subpopulation of kit foxes in the city of Bakersfield was stable despite ongoing urbanization [16, 17] and was considered a possible source for reintroductions to hedge against catastrophic declines in natural lands [17, 18]. However, after the initial detection of mange in 2013 [19], disease spread rapidly throughout this urban subpopulation causing substantial mortality. More than 460 kit foxes have been infested as of October 2018, and all cases have been lethal if not treated ([5, 19], BLC, unpublished data). In January 2019, sarcoptic mange was detected in a smaller subpopulation of kit foxes living in the city of Taft, 58 km southwest of Bakersfield (BLC, unpublished data).

Kit foxes in these unique and valuable urban populations are relatively habituated to human presence and easily captured, and affected individuals can be treated in rehabilitation facilities [19]. However, more efficient population level management, including prevention of the spread of mange, could have a greater impact than individual animal treatment. Without knowing whether kit foxes share mite variants with sympatric species such as coyotes (*Canis latrans*), dogs (*C. lupus familiaris*), or red foxes (*Vulpes vulpes*), it is not known whether management strategies to address mange in kit foxes must incorporate the other hosts as well. Accordingly, the goal for this project was to use molecular epidemiology to determine if the Taft outbreak is part of the Bakersfield outbreak and to understand the extent to which sympatric species share *S. scabiei* mite variants with kit foxes. Such insight would be invaluable, as management strategies that target multiple canids would be considerably more difficult rather than a single species strategy primarily due to varying ecology, home range size, ease of capture for application of preventative treatment and public acceptance of prevention programs (e.g. dogs *vs* coyotes) [17, 20].

## Methods

### Collection of *Sarcoptes scabiei* mites

**Fig. 1:**
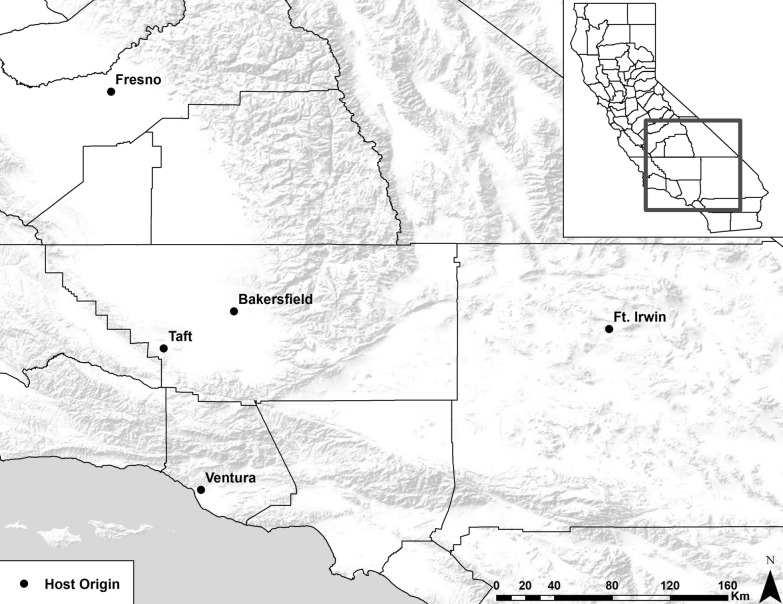
Location of mite-host sample areas in California (black dots). Hosts collected from each location included 2 dogs (*Canis lupus familiaris*), 5 coyotes (*Canis latrans*), 22 San Joaquin kit foxes (*Vulpes macrotis mutica*) and 4 red foxes (*Vulpes vulpes*) from Bakersfield, 5 San Joaquin kit foxes from Taft, a red fox from Fresno, a coyote from Ft. Irwin and a coyote from Ventura

Sampling was opportunistic, consisting of male and female animals aged 4 months and older, and that were found dead, euthanized for humane reasons, or euthanized because of threats to public safety or domestic animals. These included foxes found dead or euthanized due to mange provided by the Endangered Species Recovery Program (ESRP) and the California Department of Fish and Wildlife (CDFW), ill stray dogs from Kern County Animal Services and Bakersfield Animal Care Center, and coyotes found dead due to vehicular strike (at Fort Irwin), euthanized due to severe mange (Ventura), hit by vehicles, or euthanized for depredation by the United States Department of Agriculture Wildlife Services (USDA, Fig. 1).

All carcasses were frozen at − 20 °C and then transferred to the CDFW Wildlife Investigations Laboratory (WIL) for examination and sampling of mites. Carcasses were carefully examined for skin scaling, hair loss, pustules, papules and hyperkeratosis along the tail, rear legs, dorsum, abdomen, elbows, forelegs, neck and head. Skin at the periphery of suspected lesions was scraped with a sterile surgical blade; contents of the scraping were placed onto a clean microscope slide with 3 drops of sterile water and examined under a magnification of 40× for presence of mites. Mites were morphologically identified as *S. scabiei* [21] and 5 × 5 cm sections of mite-positive skin were excised and stored in sterile plastic bags. Because mites were rare on dog skin, positive skin scrapings from dogs were flushed from the glass slide into 2 ml microfuge tubes with 70% ethanol. Skin samples and microfuge tubes were stored at − 20 °C.

### Preparation of mite DNA and microsatellite analysis

Frozen skin and scraping contents were thawed and individual mites collected using microscopy. Each mite was pierced with a sterile 18-gauge needle under a dissecting microscope and digested overnight in lysis buffer and proteinase K (Qiagen, Valencia, CA, USA) at 56 °C. The Micro DNA Extraction Kit (Qiagen) procedure was used for the preparation of mite DNA from each individual mite according to the manufacturer’s recommendations. Final DNA from each mite was eluted in 60 µl of buffer AE.

We used 10 microsatellite markers (SARMS 33–38, 40, 41, 44 and 45) to genotype mites with modifications to the published protocol [10]. Forward primers were labelled with HEX or 6-FAM dye (Integrated DNA Technologies, Coralville, IA, USA) and reconstituted into 100 µM working dilutions. Primer pairs were combined into paired multiplexes with 1.5–2.5 µM of each primer. We performed PCR using the Qiagen 2× Type-it Multiplex PCR Master Mix, 10× multiplex primer mix (2.5 µl), DNA-free water (7 µl) and 2–3 µl DNA for a total reaction of 25 µl. Thermocycling conditions were as published [11]. PCR products were transferred to 96-well plates (Biotix Inc., San Diego, CA, USA) for electrophoresis and measurement of length polymorphisms on an ABI 3730 analyzer (Thermo Fisher Scientific, CA, USA) at the Veterinary Genetics Laboratory (Davis, CA, USA) and output sequence alignments were automated using the program STRand [22]. Microsatellite allele scoring was performed with the R-package *MSATALLELE* [23]. Data were first organized in an Excel (Microsoft, Redmond, WA, USA) spread sheet and converted into genepop format using the program CREATE [24]. Possible genotyping errors due to stuttering or large allele dropout were evaluated using MICRO-CHECKER [25]. Null alleles were estimated using ML-RELATE [26].

### Genetic analysis for population differentiation

Initially, we genotyped 20–30 mites from each host individual, except where only one or two mites could be found (e.g. dogs). After genotyping 351 mites from 19 canids at 20–30 mites per individual, it was observed that mites from individual kit fox lacked genetic diversity, therefore subsequent analysis only incorporated 1–6 mites per kit fox host individual.

For mites from each host population, we estimated allelic richness (R), number of polymorphic loci, expected (H_e_) and observed heterozygosity (H_o_), Hardy-Weinberg equilibrium (HWE), linkage disequilibrium (LD) and partitioned components of variance using analysis of molecular variance (AMOVA). To evaluate differentiation among the *S. scabiei* mite populations, we calculated the pairwise F_ST_ and visualized the differences using principal components analysis (PCA). Analyses were completed using the software GENALEX v.6.2 [27] and R packages [28] *PopGenReport* [29], *adegenet* [30] and *poppr* [31]. *P*-values ≤ 0.05 were considered statistically significant. We used a multilocus Bayesian clustering algorithm in STRUCTURE [32] to determine the number of population groups (K) and to probabilistically group individuals without using the known geographical location or host species (dog, coyote, red fox, kit fox). We used the population admixture model with a flat prior and assumed that allele frequencies were correlated among populations. We ran simulations for 800,000 iterations following a ‛burn-in’ period of 200,000. We used these initial settings to estimate the probability of one through eight clusters (K), with each run replicated 10 times. We averaged the log Pr(X|K) statistics across the multiple runs for each of the eight K estimates. We selected the K value of highest probability by identifying the set of values where the log Pr(X|K) value was maximized and subsequently selecting the minimum value for K that did not sacrifice explanatory ability [33, 34]. We defined membership to a cluster based upon the highest proportion of ancestry to each inferred cluster. Origin of hosts was mapped using ArcGIS version 10.3.1 (ESRI, Redlands, CA, USA).

## Results

We assessed population genetics of 445 *S. scabiei* mites from 41 host individuals in four host species, including six mites from two dogs from Bakersfield, 137 mites from seven coyotes (five from Bakersfield, one from Fort Irwin, and one from Ventura), 192 mites from 22 kit foxes from Bakersfield, 23 mites from five Taft kit foxes, and 87 mites from four red foxes from Bakersfield and one from Fresno (Table 1). Although occasional loci of particular mites did not amplify (Additional file 1: Table S1), 60 alleles were detected across the 10 microsatellite loci of all four host species, ranging from two alleles detected at SARM-38 to 11 alleles at SARM-33 (Additional file 2: Table S2). A total of 31 private alleles (i.e. alleles found only in one population and among the broader collective populations of study) were detected, distributed among eight loci. All loci showed LD (*P* = 0.001) and significant deviations from HWE (*P* < 0.001).

**Table 1:** Total number of alleles and the number of private alleles detected in 10 microsatellite loci for 445 *Sarcoptes scabiei* mites

Locus	No. of alleles	No. of private alleles
SARM-33	11	7
SARM-34	10	8
SARM-35	4	3
SARM-36	3	0
SARM-37	4	2
SARM-38	2	0
SARM-40	0	5
SARM-41	6	2
SARM-44	5	1
SARM-45	5	3

### Kit fox mites

Among the Bakersfield kit fox-derived mites, despite relatively large numbers of mites, there was low overall mean allelic richness (R = 1.29) and all except SARM-33, 40, 41 and 44 were monomorphic (Table 2, Additional file 1: Table S1). There were 15 alleles across the variable microsatellite loci including a private allele at SARM-40 at a frequency of 0.003. Values of H_o_ (0.082) and H_e_ (0.117) were relatively low. SARM-33, 40 and 41 were not in HWE, had possible null alleles at frequencies of 0.135, 0.177 and 0.167, respectively, and SARM-33 and 40 were in LD (*P* = 0.001). Mites collected from Taft kit foxes were similar to Bakersfield kit fox mites and also had low allelic richness (R = 1.19). There were only two polymorphic loci, SARM-40 and 44 (Table 2) and a total of 12 alleles across all loci. No private alleles were detected and, similar to Bakersfield kit fox mites, H_o_ (0.076) and H_e_ (0.085) were low. All alleles were in HWE and there was no LD.

**Table 2:** Characteristics of genetic variability among 445 *Sarcoptes scabiei* for each host-derived mite populations

Mite host	No. of mites	R	No. of polymorphic loci	H_o_	H_e_
Bakersfield kit fox (*n* = 22)	192	1.29	3	0.082	0.117
Taft kit fox (*n* = 5)	23	1.19	2	0.076	0.083
Bakersfield red fox (*n* = 4)	67	1.28	3	0.071	0.119
Fresno red fox (*n* = 1)	20	1.28	4	0.100	0.114
Bakersfield dog (*n* = 2)	6	2.08	8	0.017	0.368
Bakersfield coyote (*n* = 5)	103	2.78	10	0.245	0.520
Ft. Irwin coyote (*n* = 1)	15	1.79	7	0.288	0.273
Ventura coyote (*n* = 1)	19	1.13	2	0.023	0.032

*Abbreviations*: n, no. of hosts sampled; R, allelic richness; H_o_, observed heterozygosity; H_e_, expected heterozygosity

### Red fox mites

Red fox-derived mites from Bakersfield had multiple alleles only at the same three loci as kit fox mites, whereas SARM-36 was also variable among the Fresno red fox mites (Table 2). A single private allele was detected at SARM-41 at a frequency of 0.05 in mites from the Fresno red fox and no private alleles were detected in mites from the Bakersfield red foxes. Mean R was low for both Bakersfield and Fresno (R = 1.28) with 15 and 14 total alleles detected in these mite populations, respectively. Values of H_e_ from both Bakersfield and Fresno resembled the kit fox mites, as did H_o_ from Bakersfield, whereas H_o_ from Fresno was slightly lower (0.114). The only deviation from HWE was at SARM-33 in mites from Bakersfield (*P* < 0.05). A null allele was detected at SARM-33 at a frequency of 0.295. SARM-33 and SARM-40 were in LD (*P* = 0.04). Null alleles were not detected in mites from the Fresno red fox and there was no LD.

### Domestic dog mites

Despite obtaining only three mites from each of the two dogs, only two loci (SARM-35 and 37) had fixed alleles with a total of 22 alleles detected (Table 2). Two private alleles were detected at SARM-34. Average allelic richness was 2.08 and dog mites had lower H_o_ (0.017), but higher H_e_ (0.368) than mites collected from foxes (Table 1). Only SARM-35 and SARM-37 were in HWE and null alleles were detected at SARM-33 and 34 at frequencies of 0.382 and 0.403, respectively. LD was detected between SARM-33 and 34 (*P* = 0.003), 36 (*P* = 0.02), 38 (*P* = 0.02), 40 (*P* = 0.03); SARM-34 and 36 (*P* = 0.05), 38 (*P* = 0.04) and 40 (*P* = 0.04); SARM-36 and 38 (*P* = 0.03) and 40 (*P* = 0.03); SARM-38 and 40 (*P* = 0.02); and SARM-41 and 44 (*P* = 0.003).

### Coyote mites

Similar to dogs, the majority of loci in mites from coyotes were variable, with only three fixed loci in mites from Ft. Irwin (SARM-36, 37 and 38) and none from Bakersfield (Table 2). However, all except SARM-34 and 44 were monomorphic in mites from Ventura. Total numbers of detectable alleles were much higher than for other mite populations, with 52 detectable alleles in Bakersfield coyotes. There were private alleles in Bakersfield coyotes at eight loci (SARM-33, 34, 35, 37, 40, 41, 44 and 45), a single private allele in Ft. Irwin at SARM-40 and a single private allele in Ventura at SARM-34. Mean R (2.78) was notably high in these Bakersfield coyote mites whereas the lowest overall H_o_ (0.023) and H_e_ (0.032) occurred in the Ventura coyote mites. There was significant HWE departure for Ventura SARM-44 (*P* = 0.01) and for all loci in Bakersfield coyote mites (*P* < 0.05), but none in Ft. Irwin. Null alleles were detected at all loci except SARM-45 at frequencies ranging between 0.121 and 0.364 for Bakersfield coyote mites. Bakersfield coyote mites had LD at SARM-33 and 34 (*P* = 0.002), 36 (*P* = 0.02), 37 (*P* = 0.001), 38 (*P* = 0.001), 40 (*P* = 0.001), 41 (*P* = 0.001), 44 (*P* = 0.001) and 45 (*P* = 0.02); SARM-34 and 36 (*P* = 0.001), 38 (*P* = 0.001) and 44 (*P* = 0.002); SARM-35 and 45 (*P* = 0.001); SARM-36 and 38 (*P* = 0.001); SARM-37 and 38 (*P* = 0.001), 40 (*P* = 0.001) and 44 (*P* = 0.001); and SARM-40 and 44 (*P* = 0.001) and 45 (*P* = 0.01). LD was also detected in Ft. Irwin coyote mites between SARM-36 and 40 (*P* = 0.03).

### Host population differentiation

**Fig. 2:**
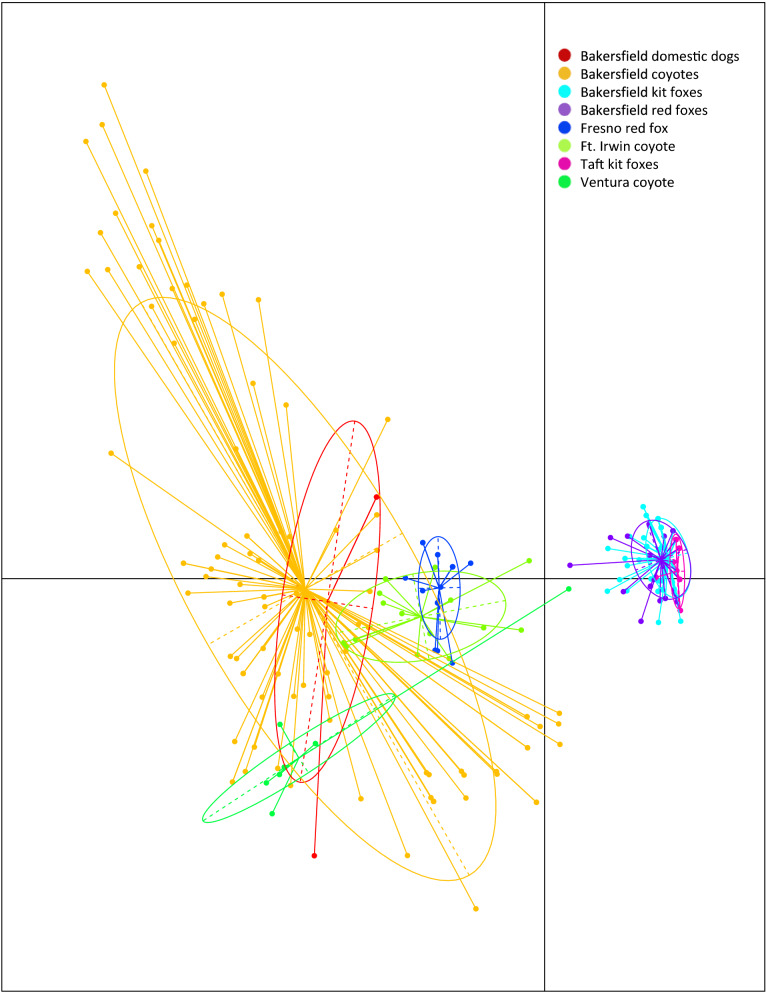
Dispersion of dog-, coyote-, kit fox- and red fox-associated mite populations according to principal components analysis (PCA). Each color-coded shape represents a single mite taken from 4 different host species

AMOVA analysis showed significant differentiation among host species-derived mite populations (46.8%, *P* = 0.001). Pairwise F_ST_ values showed the most closely related populations were mites from Bakersfield and Taft kit foxes (F_ST_ = 0.038), followed by mites from kit foxes and red foxes from Bakersfield (F_ST_ = 0.05, Table 3). The least related mites were from the Taft kit foxes and Ventura coyote (F_ST_ = 0. 843). Bakersfield kit fox and Bakersfield coyote mites were also genetically distinct (0.508) as were Bakersfield coyote and dog mites (0.168). These relationships were also clear on the scatterplot of the PCA of mites, on which Bakersfield and Taft mites clustered together, but were distinct from all other mites (Fig. 2).

**Table 3:** F_ST_ estimates for 10 microsatellite loci examined from 445 *Sarcoptes scabiei* mites for each host-derived mite population

	Bakersfield dogs	Bakersfield coyotes	Ft. Irwin coyote	Ventura coyote	Bakersfield kit foxes	Fresno red fox	Bakersfield red foxes	Taft kit foxes
Bakersfield dogs	–							
Bakersfield coyotes	0.168	–						
Ft. Irwin coyote	0.344	0.109	–					
Ventura coyote	0.626	0.263	0.553	–				
Bakersfield kit foxes	0.692	0.508	0.636	0.795	–			
Fresno red fox	0.663	0.301	0.558	0.787	0.706	–		
Bakersfield red foxes	0.657	0.413	0.563	0.796	0.050	0.701	–	
Taft kit foxes	0.704	0.407	0.627	0.843	0.038	0.781	0.123	–

**Fig. 3:**
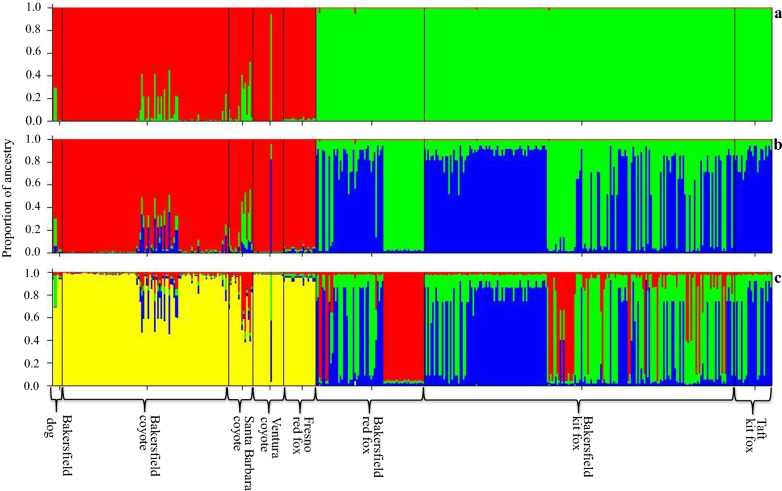
Bayesian population clustering of sarcoptic mange mites for two (**a**), three (**b**) and four (**c**) K clusters based on a multilocus microsatellite genotype dataset

Bayesian clustering of the total data set revealed maximum log Pr (X|K) for three clusters. The first cluster was composed of the mite samples from dogs, Bakersfield coyotes, Santa Barbara coyotes, Ventura coyotes and Fresno red foxes. The second group included some of the mite samples from Bakersfield red foxes and some of the mite samples from Bakersfield kit foxes. The last cluster included the remainder of the Bakersfield red fox and kit fox mite samples, as well as the Taft kit fox mite samples (Fig. 3). The data show strong separation between the group composed of mites collected from dogs, coyotes and Fresno red foxes and a second group composed of mites collected from kit fox and Bakersfield red fox. Additional structure is apparent within the sample of mites collected from kit fox and Bakersfield red fox.

## Discussion

*Sarcoptes scabiei* infests a wide array of hosts in many communities. Although multiple hosts in a community may experience mange simultaneously, examination of mite genetics often reveals various degrees of host preference and specificity, a phenomenon described as the “host-taxon law” [6, 10, 11, 35–41]. Mange is fatal in kit foxes and may contribute to local extinction of the endangered San Joaquin kit fox in Bakersfield [19, 42]. Therefore, we investigated the population genetic structure of *S. scabiei* mites among these host species in order to understand the risk that coyotes, dogs and red foxes pose for mite spillover into kit foxes, with the ultimate goal of developing an intervention strategy. Our data clearly reveal host specificity and that kit foxes acquire mite infestations from other kit foxes, both in Bakersfield and in Taft.

Based on data from 10 microsatellite markers, the most important source of population structure (46% on AMOVA) among the mites was the host and to a lesser degree geographical source, although samples from geographical locations outside of Bakersfield were limited as this was not the original intention of the study. Bakersfield mites from kit and red foxes, and mites from Taft kit foxes, had relatively few alleles and low heterozygosity compared to dogs and coyotes, comprising an obvious cluster differentiated from other host species regardless of geographical area. However, red fox mites from Fresno were less closely related to the Bakersfield or Taft fox mites, and regardless of location. While mites from domestic dogs and coyotes were intermixed, suggesting different epidemiological cycles of mange in different geographical areas, especially from areas of greater distance from Bakersfield (e.g. Fresno, Ventura and Ft. Irwin) in which contacts with mange-infested interspecific species is limited or non-existent. The lack of genetic variability and fixation of alleles among Bakersfield kit and red foxes is consistent with a founder event in the Bakersfield fox population. Because the Bakersfield outbreak was reported six years prior to the Taft outbreak, it is suspected that the source of mites in Taft was Bakersfield. Further host genetic analysis of Bakersfield and Taft kit foxes could elucidate gene flow between foxes in these locations, and additional camera or telemetry data could help clarify prospects for gene flow among the kit foxes and mites as well.

Thirteen of the 14 alleles detected in Bakersfield kit fox mites, all 12 alleles in Taft kit fox mites, and all 15 alleles in red fox mites from Bakersfield were also found in coyotes and domestic dogs. The Bakersfield kit fox-derived mites had one private allele, but only at low frequencies which could be due to genotyping error. Presence of private alleles can indicate isolation of mite populations and host-associated genetic variants [11]. *Sarcoptes scabiei* mites can be transmitted between red foxes and domestic dogs in Europe [10], supporting the likelihood that the original source of mange among Bakersfield kit foxes was coyotes or dogs. Further indication of an original spillover in Bakersfield and Taft with a founder event is the absence of polymorphic loci across 282 mites collected from 31 foxes (27 kit foxes and 4 red foxes) belonging to the same genus (*Vulpes*). Bakersfield dog and coyote mites had high R, H_o_ and H_e_ consistent with a large ancestral population of mites as is described for other mites collected from the genus *Canis* [12, 43].

Multiple studies [11, 13, 40, 41, 43] have examined a relatively small number of mite individuals from a host individual, implicitly assuming that genetic variability of mites on a single host was negligible. We found little benefit to sampling more than 25–30 individual mites per host individual in our study, in part because common alleles tend to be more informative than rare alleles when evaluating genetic composition within a population [44], and also because there was so little genetic variability among mites from any kit fox individual. Moreover, in the case of dogs, we could not necessarily achieve our target sample size of 30 mites. Despite this limitation, the six mites we did collect from two dogs showed considerable heterozygosity and allelic richness.

Coyote mites from Bakersfield, while the most variable, also had significant deviations from HWE, were in LD, and may have had null alleles, findings similar to other *Sarcoptes* and mite studies [10, 43, 45]. Null alleles are found in most taxa and are especially prominent in insects and bivalves, resulting in homozygous excess and deviations from HWE [46–48]. This suggests that we may be underestimating the total variability in the coyote mite population. Given the larger home range sizes of coyotes, it is also possible that the deviations from HWE could have occurred if locations of sampling of coyotes and their mites were different from the areas where they typically resided (e.g. Wahlund effect).

Microsatellites are commonly used to study recent evolutionary events and have often been used to study molecular genetics of mange, thus our use of microsatellites allows our results to be compared directly to findings in the literature. There are other molecular methods such as Radseq, which analyzes single nucleotide polymorphisms (SNPs), which may offer insight into finer scale phylogeographical patterns [49] and possible historic host-associated introductions. Further, use of SNPs could potentially avoid some of the biases and subjectivity that can impact allele scoring of microsatellites. Whether using microsatellites or whole next-generation genotypical approaches, ongoing genetic surveillance will be necessary to detect rare cross-species transmission among southern San Joaquin Valley canids which could initiate mange emergence in exurban kit foxes, an event that could contribute to species wide declines.

Scabies in humans is often associated with overcrowding such as in hospitals, nursing homes, prisons and schools [50–54]. High population densities may underlie sarcoptic mange epidemics in wildlife as well [55]. The Bakersfield kit fox population is uniquely dense relative to exurban populations and these kit foxes may share dens with skunks (*Mephitis mephitis*) and domestic cats (*Felis catus*) or occupy dens previously used by red foxes, coyotes, or feral dogs. Such a complex contact network with high density and high host biodiversity could exacerbate the mange epidemic; however, biodiversity *per se* appears unlikely to contribute given our finding of host-restricted mite variants. The high densities of Bakersfield kit foxes may contribute to the severe impact of mange on this population, which could suggest that less dense exurban populations may be at less risk. The existence of host-restricted mite variants could also explain why it is common to observe mange-infested animals living in a community with other, mange-free host species.

## Conclusions

Large epidemics of sarcoptic mange with high case fatality rates can devastate free-ranging wildlife [56–60]. Spillover of mites from common and less clinically impacted species such as coyotes and domestic dogs, poses an important conservation challenge for wildlife managers and can inhibit recovery efforts [61]. Therefore, documenting the true likelihood of acquiring infestation from conspecific and sympatric hosts is crucial for intervention to support the most at-risk species. Our data clearly document that kit fox mites circulate primarily among Bakersfield kit foxes with occasional transmission or spillover to and from red foxes. The recent Taft epidemic is closely related to the epidemic in Bakersfield kit foxes and probably derived from Bakersfield kit foxes directly. This suggests that efforts to control mange in the kit fox population should focus on kit foxes and that other species are not primarily involved in this epidemic.

## Supplementary information

**Additional file 1: Table S1.** Raw microsatellite genotypes of the 10 SARM microsatellite loci for *Sarcoptes scabiei* mites. A total of 445 mites were collected from the skin of 2 dogs (*Canis lupus familiaris*), 5 coyotes (*Canis latrans*), 22 San Joaquin kit foxes (*Vulpes macrotis mutica*) and 4 red foxes (*Vulpes vulpes*) from Bakersfield, CA. An additional 59 mites were collected from 5 kit foxes in Taft, a red fox from Fresno, a coyote from Ft. Irwin and a coyote from Ventura, CA. Data are organized by mite-derived host and host location.

**Additional file 2: Table S2.** Distributions of allele frequencies in 10 microsatellite loci among *Sarcoptes scabiei* mite populations by host and host location (allele sizes are in base pairs). Private alleles are denoted with “†”.

## Data Availability

Datasets supporting the findings of this article are included in the article and its additional files.

## Abbreviations

H_e_: expected heterozygosity
H_o_: observed heterozygosity
HWE: Hardy-Weinberg equilibrium
LD: linkage disequilibrium
*n*: number of hosts sampled
PCA: principal components analysis
R: allelic richness
SARM: *Sarcoptes* microsatellite locus
